# Tocilizumab for critically ill patients with refractory anti-NMDAR encephalitis: a case report

**DOI:** 10.3389/fimmu.2026.1809134

**Published:** 2026-09-01

**Authors:** Renato Pierre Lima, J. H. F. Ferreira, Marcos Vinícius Tadao Fujino, Ana Luíza Vieira de Araújo, Mauricio Elias Nunes da Silva, Lívia Almeida Dutra

**Affiliations:** Instituto de Ensino e Pesquisa, Hospital Israelita Albert Einstein, São Paulo, SP, Brazil

**Keywords:** anti-N-methyl-D-aspartate receptor encephalitis, autoimmune encephalitis, ICU, rituximab, tocilizumab

## Abstract

**Background:**

Anti-*N*-methyl-D-aspartate receptor (anti-NMDAR) encephalitis often requires intensive care and rapid escalation of immunotherapy. We report a critically ill woman with refractory anti-NMDAR encephalitis treated with tocilizumab (TCZ) as early second-line therapy and discuss the rationale for IL-6 receptor blockade over other treatment strategies.

**Case report:**

A previously healthy 30-year-old woman developed psychosis, catatonia, dyskinesias, and refractory status epilepticus; anti-NMDAR antibodies were detected in the CSF using a commercial fixed cell-based assay. She required mechanical ventilation and received methylprednisolone and IVIG 35 days after symptom onset, without improvement. The course was complicated by sepsis, dialysis-dependent acute kidney injury, anemia, and thrombocytopenia. Given active infection, cytopenias, and prior hepatitis B exposure, TCZ was initiated with tenofovir prophylaxis. Over the subsequent 30 days, she improved, was extubated, and became seizure-free. Rituximab (RTX) was started 9 months after onset. At 25-month follow-up, outcomes were mRS 2, CASE 1, and MoCA 26/30.

**Conclusion:**

This case describes rapid clinical improvement temporally associated with TCZ administration in a critically ill patient with refractory anti-NMDAR encephalitis. Although causality cannot be established, TCZ may offer a rapid and time-limited immunomodulatory option for carefully selected ICU patients when conventional second-line therapies are unsuitable. Prospective controlled studies are required to determine its efficacy and optimal timing.

## Introduction

Anti-*N*-methyl-D-aspartate receptor (anti-NMDAR) encephalitis is the most common type of autoimmune encephalitis (AE). It mostly affects children, adolescents, and young adults, with a female predominance (1). Most patients present with prominent neuropsychiatric symptoms, followed by seizures, aphasia, and reduced level of consciousness (2). Up to 77% require intensive care unit (ICU) admission (1), and a substantial proportion develop severe autonomic dysfunction, status epilepticus (SE), and sepsis and require mechanical ventilation (3). Prognosis in anti-NMDAR encephalitis correlates with markers of acute severity, including ICU admission, cerebrospinal fluid (CSF) pleocytosis, MRI abnormalities, and treatment delay longer than 30 days (4).

Treatment recommendations are based primarily on observational studies and expert consensus. First-line therapy comprises corticosteroids, intravenous immunoglobulin (IVIG), plasma exchange, and tumor resection when applicable (6). Approximately 50% of patients fail to improve and are subsequently treated with rituximab (RTX) and/or cyclophosphamide (CYC) (1, 7). Third-line options for refractory disease include tocilizumab (TCZ) and bortezomib. Although randomized clinical trials of second-line therapies are lacking, observational and real-world data suggest that RTX is associated with better functional outcomes and fewer relapses (8, 9). However, the use of RTX and other biologic therapies in critically ill patients raises concerns because of their prolonged immunological effects and the risk of serious infectious complications (10).

We describe a young patient with refractory anti-NMDAR encephalitis who developed severe ICU complications and was treated with TCZ as second-line therapy. We discuss the rationale for treating refractory disease and the potential advantages of TCZ over RTX and CYC in critically ill patients with severe anti-NMDAR encephalitis.

## Case report

A previously healthy 30-year-old woman presented with a 4-day history of nausea and vomiting. She subsequently developed aphasia and experienced two tonic–clonic seizures. Initial brain MRI and CSF findings were unremarkable. One week later, she developed psychosis and catatonia and was admitted to a psychiatric ward. Over the following 14 days, seizure frequency increased, orofacial dyskinesias developed, and she was transferred to the ICU. She required intubation and received methylprednisolone and IVIG 35 days after symptom onset, without clinical improvement. She subsequently developed refractory status epilepticus, acute kidney injury requiring hemodialysis, segmental pulmonary embolism, and sepsis caused by carbapenemase-producing *Klebsiella pneumoniae* (KPC). At this point, she was transferred to our service.

On initial examination at our institution, she was sedated and mechanically ventilated, with no eye contact or verbal response. Pupils were equal and reactive to light, and prominent orofacial dyskinesias were observed. Deep tendon reflexes were normal, and muscle tone was globally decreased. Laboratory tests showed anemia (hemoglobin, 9.0 g/dL), thrombocytopenia (46 × 10³/µL), elevated creatinine (2.52 mg/dL), and normal serum immunoglobulin levels. Screening for latent tuberculosis was negative, and serology was consistent with prior hepatitis B exposure (positive total anti-HBc). EEG showed extreme delta brush. Brain MRI remained normal, while CSF analysis revealed pleocytosis (21 cells/mm³) and a protein level of 21 mg/dL. A commercial fixed cell-based assay (CBA; EUROIMMUN, Lübeck, Germany) confirmed the presence of anti-NMDAR antibodies in both serum and CSF. Paraneoplastic investigation with whole-body CT and pelvic MRI was negative.

**Figure 1:**
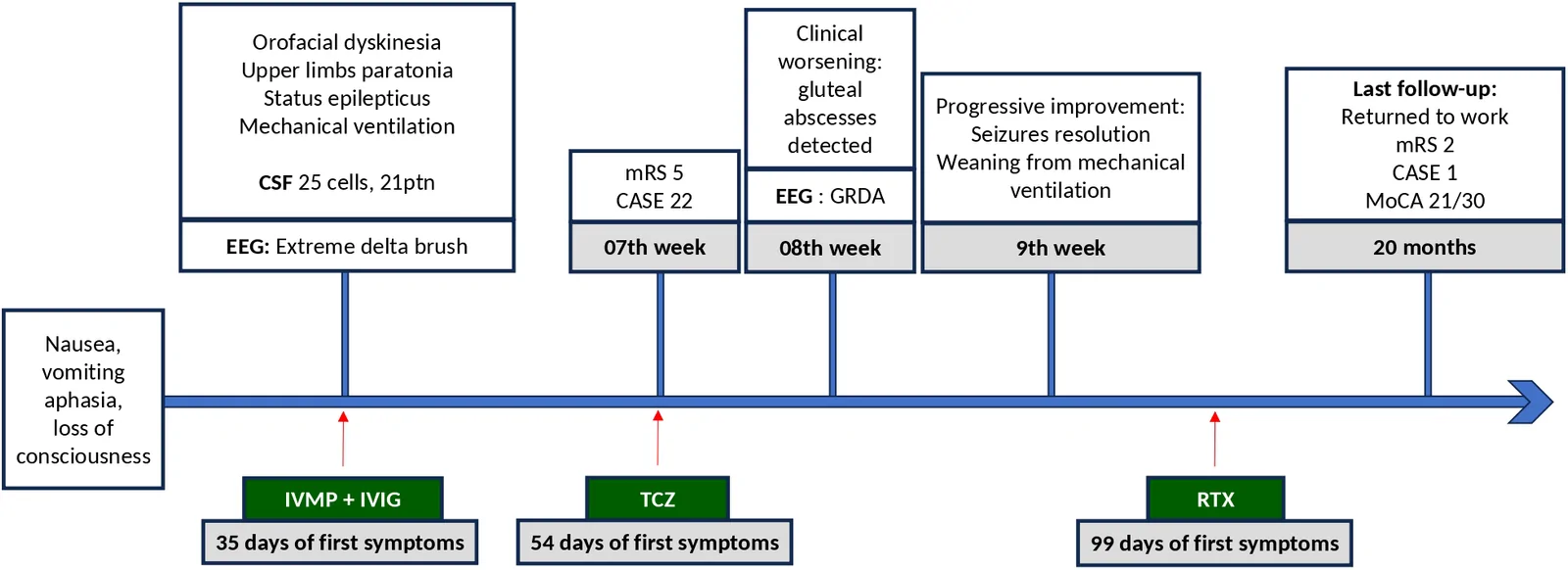
Timeline of clinical features, investigations, and treatments. CSF, cerebrospinal fluid; MPIV, intravenous methylprednisolone; IVIG, intravenous immunoglobulin; mRS, modified Rankin scale; CASE, Clinical Assessment Scale in Autoimmune Encephalitis; MoCA, Montreal Cognitive Assessment; EEG, electroencephalogram; GRDA, generalized rhythmic delta activity.

Because of the multiple clinical complications, active severe infection, and risk of hepatitis B reactivation, TCZ was initiated at 8 mg/kg monthly, together with tenofovir 300 mg/day. At that time, she was bedridden, with a modified Rankin scale (mRS) score of 5 and a Clinical Assessment Scale in Autoimmune Encephalitis (CASE) score of 22; the Montreal Cognitive Assessment (MoCA) could not be performed. One week after TCZ administration, gluteal abscesses caused by *Enterococcus faecalis* were identified and treated with teicoplanin. Over the subsequent 30 days, she showed gradual clinical and neurological improvement without further systemic complications. She was successfully weaned from mechanical ventilation and remained seizure-free. RTX was started 9 months after initial symptoms, with a washout period of 30 days from the last TCZ dose. **Figure 1** summarizes the clinical features, investigation, and treatment of the case report.

At the first outpatient follow-up, 4.6 months after symptom onset, the patient remained bedridden because of lower-limb weakness and contractures (mRS 5). Seizures remained controlled, alertness was preserved despite absent verbal output, and dyskinesias had resolved (CASE 12). At approximately 25 months after symptom onset, she showed marked functional improvement (mRS 2) and had returned to work, although with reduced performance and limited leisure activities. Cognitive assessment yielded a MoCA score of 26/30, with deficits predominantly in executive function and language. The CASE score was 1 because of mild right lower-limb weakness.

## Discussion

We report a young patient with severe anti-NMDAR encephalitis who received TCZ as an early second-line therapy. The choice was made after careful risk–benefit evaluation, due to multiple clinical complications and prior hepatitis B exposure, which is a relative contraindication to RTX. Nonetheless, this case provides an insightful discussion on the rationale for the use of TCZ over RTX or CYC in the ICU.

First, we had a patient with refractory disease, requiring rapid immunomodulation and with severe clinical complications such as KPC active infection, cytopenias, and renal replacement therapy, which substantially increased the hazards of immunosuppression. Second, RTX requires more than 30 days for its peak clinical effect, usually requires two doses with an interval of 14 days, and leads to sustained peripheral B-cell depletion for 6–12 months, which may be a problem in the ICU environment. Third, patients with severe anti-NMDAR encephalitis receiving RTX frequently require additional IV CYC, at least in the first month, exactly because of the latency for RTX effect, which also poses a problem in critically ill patients.

Alternatively, CYC is an alkylating agent that induces rapid, non-selective immunosuppression through broad lymphocyte depletion; however, its use was considered unsafe due to the risk of worsening cytopenia (myelosuppression) and infectious complications (5). Notably, clinical trials on RTX or CYC for AE are lacking, and most available evidence comes from case reports, series, and real-world data (6–8), complicating the assessment of therapeutic benefit relative to potential harm.

Conversely, TCZ is an IL-6 receptor-blocking monoclonal antibody, with action-onset effect as early as 2 weeks and a half-life close to 3 weeks (11), that could be administered IV or subcutaneously. We have data on the use of TCZ in the ICU, derived mostly from the COVID-19 pandemic, where it was associated with improved clinical outcomes and survival in patients with hypoxia and systemic inflammation (12). Common adverse effects of tocilizumab include infections, elevated liver enzymes, gastrointestinal symptoms, headache, and rash; but serious events such as severe liver injury or hypersensitivity reactions are rare. Thus, TCZ has not been associated with a significant increase in serious adverse events in ICU settings, including patients with multiple comorbidities and on mechanical ventilation, and the risk of myelosuppression or hepatitis B reactivation is lower than RTX and CYC (5).

At the moment, TCZ is currently recommended as rescue third-line therapy for refractory AE (6, 13), and observational data suggest potential benefits. In a single-center cohort of 91 AE patients with inadequate clinical response to first-line immunotherapy followed by RTX, escalation to TCZ (*n* = 30) was associated with a higher rate of favorable mRS outcomes at 2 months and at last follow-up (14). Additionally, in a prospective cohort of anti-NMDAR encephalitis, completing the T-SIRT sequence (teratoma removal, steroids, IVIG, RTX, and TCZ) within 1 month of symptom onset correlated with greater 1-year functional outcomes, supporting early escalation that includes tocilizumab in selected patients (15).

Beyond autoimmune encephalitis, growing experience with TCZ in MOGAD, cryptogenic NORSE/FIRES, pediatric neuroinflammatory disorders, and neuromyelitis optica spectrum disorder has expanded the safety data on IL-6 receptor blockade in neurological diseases (16–20). In our patient, TCZ was initiated 55 days after symptom onset and was followed by neurological improvement within 1 week and complete seizure control within 2 weeks. This temporal association, together with the need for rapid and time-limited immunomodulation, supported the rationale for selecting TCZ in this particularly complex ICU setting.

Nevertheless, this report has the inherent limitations of a single-case design. The clinical improvement cannot be attributed exclusively to TCZ, as spontaneous disease stabilization and a delayed effect of methylprednisolone or IVIG cannot be excluded. Furthermore, these findings cannot be generalized or used to determine the efficacy or optimal timing of TCZ. Prospective controlled evidence is therefore required. In this context, the ongoing phase 3 randomized CIELO trial is evaluating the efficacy and safety of satralizumab, another IL-6 receptor inhibitor, in patients with anti-NMDAR or anti-LGI1 encephalitis and may provide evidence to guide future treatment decisions (21).

In summary, this case illustrates the therapeutic challenges of managing severe anti-NMDAR encephalitis in the ICU when conventional second-line therapies may be unsuitable because of active infection, cytopenias, renal failure, or the risk of hepatitis B reactivation. TCZ may represent a rapid and time-limited immunomodulatory option for carefully selected critically ill patients; however, its role as an early second-line therapy requires confirmation in prospective controlled studies.

## Data Availability

The raw data supporting the conclusions of this article will be made available by the authors, without undue reservation.
